# Timing Decomposition and Strategy Trade-Offs in Contrast Detection Autofocus Under Platform Capability Constraints

**DOI:** 10.3390/s26123770

**Published:** 2026-06-12

**Authors:** Ximing Zhang, Rui Hai, Yulin Wang, Weiping Liu

**Affiliations:** College of Instrumentation and Electrical Engineering, Jilin University, 938 West Democracy Avenue, Changchun 130061, China; zhangxm21@mails.jlu.edu.cn (X.Z.);

**Keywords:** contrast detection autofocus, industrial machine vision, platform capability constraints, frame-level transaction chain, latency decomposition, controlled perturbation injection, performance envelope, strategy trade-offs

## Abstract

**Highlights:**

**What are the main findings?**

- On the industrial black-box platform, the dominant observable tail lies after control submission, in the command-to-actuation segment.
- Controlled one-factor perturbations on the bridging platform reproduce the main degradation directions observed on the industrial black-box target, associated with sample position mismatch and actuation chain variability.

**What are the implications of the main findings?**

- Contrast detection autofocus performance should be compared under explicit platform capability constraints, with tail risk interpreted jointly through peak detection, post-decision–actuation, and post-peak repositioning.
- Capability tiering, segment-wise bottleneck localization, and strategy-frontier analysis link deployment, platform evolution, and evaluation to measured capability conditions rather than to device labels.

**Abstract:**

Contrast detection autofocus (CDAF) performance in industrial machine vision is shaped by platform capability as well as by the focus measure and search strategy. CDAF is analyzed through a platform capability framework and a unified frame-level transaction chain across three platforms: a capability upper-bound platform (P1), a bridging platform (P2), and an industrial black-box platform (P3). In experiments covering six scene categories, four initial conditions, five fixed-rule strategies, and 30 repetitions per condition, the dominant observable tail on P3 is localized after control submission, in the command-to-actuation segment. On P2, controlled one-factor perturbations using a physically calibrated sample position mismatch intensity (σ_align_) and an actuation chain variability coordinate (λ_act_) reproduce the main P3 degradation directions, providing a mechanism-level account in terms of sample position mismatch and command-to-actuation variability. Platform capability sets the reachable performance boundary, within which strategies trade speed, final quality, and failure risk. On P3, S1–S4 form the main engineering trade-off band, whereas S5 shows condition-dependent upper-quantile quality gains without a stable frontier advantage. The resulting deployment logic combines capability tiering, segment-wise bottleneck localization, and strategy band selection and treats CDAF as a capability-conditioned speed–quality–risk trade-off rather than a platform-independent strategy ranking.

## 1. Introduction

In industrial machine vision, contrast detection autofocus (CDAF) is conventionally formulated as a focus measure and search strategy problem, and comparative surveys have organized existing algorithms along these two axes [1]. Recent autofocus work has continued to broaden focus quality indicators, including transform-domain and contrast-related measures [2], while the focus measure side has examined operator selection under unimodality and noise robustness criteria [3] and provided systematic operator-level analyses including computational cost [4]. Subsequent work has further proposed robust focus measures for difficult imaging conditions such as low-contrast scenes [5]. On the search strategy side, industrial autofocus algorithms have addressed peak crossing detection, overshoot suppression, and autofocus speed evaluation [6], typically under the implicit assumption that the imaging-and-actuation chain provides timely and predictable feedback. Quantitative evaluation protocols for focus measure operators in microscopy and machine vision contexts have also been developed [7]. In deployed systems, however, the timing relationship among image acquisition, control submission, and actuator response can be equally decisive—a dimension less directly addressed by focus measure or search strategy design alone.

From a deployment perspective, CDAF is not only an offline focus curve search but also a sampled frame-driven control loop. In one update, the optical state is sampled during exposure, transported through readout, buffering, and host callback, converted into a focus measure and a control command, and finally applied by the focusing actuator. If the sampling, computation, command transfer, or actuation stages are delayed or non-deterministic, the focus value used for a decision can correspond to an earlier actuator position, and a stop or rollback command can take effect only after additional post-peak motion. Related studies have further extended autofocus systems through region-of-interest (ROI)-adaptive control [8], high-speed focal sweep [9], visual servoing [10], illumination-assisted focusing [11,12,13], event-driven sensing [14], and lens- or sensor-level focusing hardware [15,16]. These studies indicate that available timing markers, feedback structures, illumination routes, and actuation interfaces can reshape autofocus behavior, but such assumptions are rarely reported through a common platform capability vocabulary.

Here, CDAF is treated as a continuous frame-driven control process in which platform capability affects sampling, state judgment, control submission, and actuation onset. From the controller viewpoint, an industrial black-box platform is seen through limited event time visibility, restricted position observability, and a partly predictable actuation path. Comparisons based only on offline focus curves or isolated successful runs cannot account for the long-tail latency, overshoot, and failure risk observed in deployment.

The analysis adopts a unified view of platform capability, timing chain, and strategy trade-offs. Three platforms serve as anchors in this capability space: P1 provides an upper-bound reference under high observability, high controllability, and a near-deterministic timing chain; P2 provides a controllable bridge for uncertainty injection under repeatable logic; and P3 represents the industrial black-box deployment condition.

### 1.1. Autofocus Method Families and Implicit Platform Assumptions

Existing CDAF methods can be grouped into several representative fixed-rule families, including continuous sweep with stop-after-peak detection, stop-after-peak detection followed by fixed rollback, bidirectional bracketing with midpoint selection, coarse-to-fine two-stage search, and local hill climbing [1,6]. Continuous focal sweep autofocus has also been studied under high-speed view adjustment and more open hardware timing assumptions [9]. More open event time marking, triggering, and state-observation interfaces can support delay-aware or event-driven variants, but these variants still depend on the timing and actuation assumptions available on the platform [14].

For example, stop-after-peak detection requires samples that still represent the local focus state near the current actuator position, and it also requires the actuation path to respond within a bounded and reasonably predictable delay [6,9]. Fixed rollback assumes that the stopping lag is statistically compensable by a preset reverse displacement [6]. Bidirectional bracketing requires samples from both sides of the peak and benefits from controllable reversal or reliable position information [1]. Coarse-to-fine search relies on informative coarse samples before a finer local scan is executed [1,9]. Hill climbing requires locally informative feedback and a stable relation between actuator motion and focus value change; it is therefore sensitive to low contrast, false local peaks, and weak local monotonicity [5,10].

The later strategies S1–S5 are representative fixed-rule instances rather than an exhaustive taxonomy. They are used here to test how the prerequisites summarized above hold, weaken, or fail as timing observability, position observability, and actuation controllability change, with the platform capability variables defined in Section 2.1.

Accordingly, Table 1 is used as a compact background map: it does not attempt an exhaustive taxonomy, but links each method family tested or discussed in this paper to the platform prerequisite, typical failure mode, and representative references most relevant to the present capability analysis.

### 1.2. Research Positioning, Gap, and Contribution Boundary

Existing studies have evaluated focus measure operators and their robustness in microscopy or machine vision settings [4,7]. ROI-adaptive autofocus has been studied through dynamic region selection [8]. Microscope autofocus has also been combined with visual servo control to close the loop between image feedback and motion control [10]. Illumination-assisted autofocus has been investigated using oblique illumination, laser illumination, and laser-spot-array methods [11,12,13]. Event-camera-based autofocus provides an event-driven sensing route for microscopic imaging [14]. Liquid lens and in-sensor focusing studies show that actuator and sensing hardware can reshape autofocus dynamics [15,16]. Continuous focal sweep under high-speed view adjustment further illustrates that autofocus behavior depends on the available hardware timing and actuation assumptions [9]. Taken together, these studies show that CDAF background is not limited to the focus measure or search rule: ROI selection, visual feedback, illumination design, event sensing, and focusing hardware can all alter the operating assumptions of autofocus. What remains less explicit is a common way to state which platform capability conditions support a reported strategy advantage, and how strategy degradation and conclusion transfer should be described when position observability, actuation controllability, and timing determinism change together.

The work is therefore positioned at the platform capability and timing chain level. The focus measure and tested fixed-rule strategy set are held fixed so that changes in CDAF behavior can be attributed to observability, controllability, and timing determinism.

The contributions are fourfold. First, the paper establishes a two-dimensional platform capability framework with an actuator-side capability axis A, an imaging/chain-side capability axis C, and the capability triplet <Z, U, T> for position observability, actuation controllability, and timing observability and determinism. Second, it defines a platform-independent TS0–TS4 frame-level transaction chain and associated timing quantities, including the sampling instant (t_s_), arrival-to-effectuation latency (T_pipe_), and sample-to-actuation latency (T_sa_), to support timing comparison under a common event convention. Third, it introduces replay-based controlled perturbation injection on P2, where σ_align_ represents sample position mismatch and λ_act_ represents actuation chain variability, and compares the resulting degradation patterns with the P3 target through goodness-of-fit metrics. Fourth, it connects the P3 strategy frontier results to engineering use by linking capability tiering and dominant segment localization to strategy band selection and platform improvement priorities.

The analysis is limited to CDAF timing chain behavior and engineering trade-offs under platform capability constraints. It does not define a universal taxonomy for all autofocus systems or address full autofocus, autoexposure, and auto-white-balance coordination. ROI, focus measure (FM), and strategy set are fixed throughout the experiments.

## 2. Materials and Methods

To make the relation between the methodological components explicit, Section 2 is organized as follows: Section 2.1 defines platform capability through the <Z, U, T> tiering rule. Section 2.2 defines the TS0–TS4 frame-level transaction chain and the timing quantities used for latency decomposition. Section 2.3 specifies the engineering roles of P1, P2, and P3. Section 2.4 defines the scene matrix, tested fixed-rule strategies, performance metrics, and goodness-of-fit metrics. Section 2.5 then describes the replay-based controlled perturbation procedure used on P2.

### 2.1. Platform Capability Framework

Platform constraints are represented in a two-dimensional capability space rather than by device labels, interface names, or implementation style. The A-axis describes actuator-side capability, mainly position observability and actuation controllability. The C-axis describes the timing capability of the imaging–computation–control chain, including event visibility, chain decomposability, and temporal determinism. This capability space description identifies the platform conditions under which a given strategy can operate as assumed.

P1, P2, and P3 occupy three representative positions in this capability space, denoted as A1 × C1, A2 × C2, and A3 × C3, where the subscripts 1, 2, and 3 denote successively lower capability tiers along each axis (1 = H, 2 = M, 3 = L). P1 provides the upper-bound reference under high observability, high controllability, and a near-deterministic timing chain. P2 provides a controllable bridge on which uncertainty can be injected while the control logic remains repeatable. P3 represents the industrial black-box target, with limited interfaces, incomplete internal visibility, and a variable actuation path. The three platforms are treated as capability space anchors rather than isolated hardware setups.

A capability triplet <Z, U, T> is used to summarize the relevant properties of each capability cell: Z denotes position observability, U denotes actuation controllability, and T denotes timing observability and determinism. The L/M/H levels (low, medium, and high tiers) do not report experimental performance. They specify whether the observation, control, and timing conditions required by a strategy are available on the target platform. Under this framework, absolute performance values measured on P1 or P2 are not extrapolated to P3; what transfers are relative trends and the capability conditions under which they hold.

Operational quantitative tiering of the capability triplet. The <Z, U, T> levels are assigned using a task scale timing rule. The physical reference is the minimum effective focus displacement Δz_min_, defined as the smallest actuator displacement that produces a distinguishable focus state change outside the optical depth-of-field/focus tolerance range. In the present system, Δz_min_ is approximately 0.02 mm, estimated from measured focus response and lens depth-of-field/Gaussian optics considerations. The reference time T_ref_ is the time required for the actuator to travel Δz_min_, which is approximately 2–5 ms for the current actuator. The tier boundaries are set to 0.2 ms for the high-capability boundary and 5 ms for the upper boundary of the medium tier. For Z, U, and T, the tier is assigned by the p99 value of the relevant feedback, command-to-effect, or event timing uncertainty: H if the required state/event is available and p99 ≤ 0.2 ms, M if it is available and 0.2 ms < p99 ≤ 5 ms, and L if the required state/event is unavailable or p99 > 5 ms. The current assignments are P1 ≈ <H, H, H>, P2 ≈ <M, M, M>, and P3 ≈ <L, L, L>, as summarized in Table 2. Throughout this paper, τ_X,p99_ (X ∈ {Z, U, T}) denotes the p99 of the relevant feedback, command-to-effect, or event timing uncertainty along the X axis.

**Figure 1:**
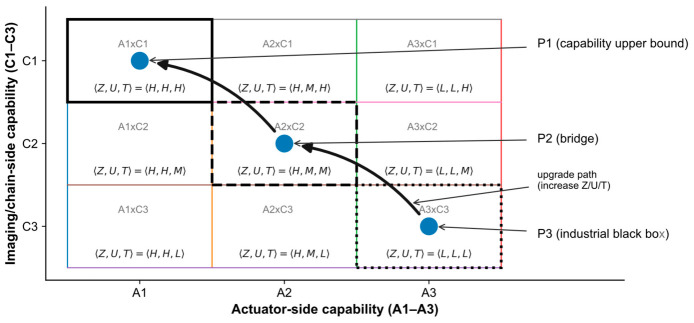
Platform capability grading and paradigm mapping. The horizontal axis is the A-axis (actuator-side capability), and the vertical axis is the C-axis (imaging/chain-side capability). The capability triplet <Z, U, T> in each cell denotes position observability, actuation controllability, and timing observability and determinism, respectively; the L/M/H tiers are assigned according to the Δz_min_- and T_ref_-based quantitative rules in Table 2. P1, P2, and P3 correspond to the capability upper-bound platform, the bridging platform, and the industrial black-box platform, respectively. The arrows indicate directions of capability improvement and bridging-based validation. Blue circles denote the three platform anchors, black rectangles highlight the representative capability regions, and the colored grid lines are used to distinguish the capability cells.

The platform capability framework, the anchor positions of P1–P3, and the direction of capability improvement are shown in Figure 1.

### 2.2. Unified Frame-Level Transaction Chain and Timing Metrics

A platform-independent frame-level transaction chain is defined to provide a common timing convention across heterogeneous platforms. Five logical events describe one sample-driven control update in CDAF: TS0 is exposure start; TS1 is the time at which the current frame becomes available in the computation domain; TS2 is completion of the focus measure computation; TS3 is commitment of the control transaction generated from that frame; and TS4 is the onset of the corresponding actuator effect. These definitions do not reconstruct every internal implementation detail. They provide a common event semantics for latency decomposition, sample position mismatch analysis, and strategy interpretation.

To distinguish the image sampling instant from the frame arrival instant, the sampling instant is defined as the exposure midpoint, t_s_ = TS0 + T_exp_/2, where T_exp_ is the exposure duration of the current frame. On this basis, two total latency quantities are defined. The first is the composite latency from the arrival to effectuation, T_pipe_ = TS4 − TS1, which characterizes the total timing chain length from the moment the frame becomes computationally available to the moment the control action truly begins to act. The second is the sample-to-actuation latency, T_sa_ = TS4 − t_s_, which characterizes the physical delay between the sampled optical state and the onset of the actuation based on that sample. The former is suited to unified cross-platform decomposition, whereas the latter is more directly related to overshoot, sample focus state mismatch, and stop compensation capability.

To explain the origin of the total latency, T_pipe_ is further decomposed into three subsegments: TS2−TS1, TS3−TS2, and TS4−TS3. TS2−TS1 represents the processing time from frame availability to completion of the focus measure. TS3−TS2 represents the time for control update and transaction commitment. TS4−TS3 represents the combined latency from command commitment through interface transfer and lower-level handling to the actual onset of actuator motion. On an industrial black-box platform, the dominant degradation does not necessarily distribute evenly across these subsegments. The decomposition is therefore used not only for statistical description, but also for localization of tail amplification.

Although the logical and control meanings of TS0–TS4 are platform-independent, their observability differs across platforms. Under P1 and some P2 conditions, more complete lower-level event time markers allow TS0–TS4 and T_sa_ to be defined relatively strictly. Under P3, however, TS1 is typically observable only after the host-side frame callback or frame grab completion, so the earlier delay from the exposure end to computational availability cannot be observed in a manner directly comparable with P1/P2. For this reason, the main cross-platform comparison is based on T_pipe_ and its segment-wise decomposition, whereas T_sa_ is primarily used on platforms that provide a strict sampling reference.

The P3 observability boundary is handled by separating three timing quantities. First, T_pipe_ = TS4 − TS1 and its segments (TS2−TS1, TS3−TS2, TS4−TS3) are observable on all three platforms under the same convention, and all segment-wise dominance analyses are based on T_pipe_. Second, T_sa_ = TS4 − t_s_ is strictly defined only on P1 and P2, because it requires t_s_ = TS0 + T_exp_/2 and TS0 is not directly time-stamped on P3. Third, the unobservable front-end offset on P3 is denoted as Δ_hidden_ = TS1 − t_s_, with T_sa_ = Δ_hidden_ + T_pipe_. Using the observed lower bound of comparable P2 frame arrival segments (p99 ≈ 1.3 ms) and a conservative sensitivity scan upper-bound for the unobservable camera/host front-end offset under non-real-time operation (up to ≈50 ms), Δ_hidden_ ∈ [1, 50] ms is used as a plausible range. Section 3.2 reports a sensitivity scan over this range to evaluate whether the T_pipe_ tail dominance conclusion remains valid when the unobservable offset is folded into T_sa_.

**Figure 2:**
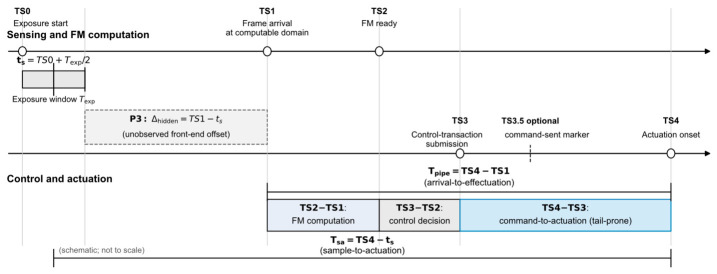
Frame-level transaction chain and key timing definitions for contrast detection autofocus. TS0–TS4 denote exposure start, frame arrival at the computable domain, completion of focus measure computation for the current frame, control transaction submission, and actuation onset, respectively. Along this event chain, the composite latency T_pipe_ from arrival to effectuation and the effective sample-to-actuation mismatch T_sa_ from sampling to effectuation are defined.

The unified frame-level transaction chain is illustrated in Figure 2, and the cross-platform observation mapping of the logical events is summarized in Table 3.

### 2.3. Experimental Platforms and Optical Chains

The experiments are organized around three platform types. P1 provides the capability upper-bound reference with high temporal determinism and high observability. P2 provides the bridging environment for controlled uncertainty injection under repeatable control logic. P3 is the industrial black-box target for deployment-side evaluation. For the perturbation scans, P2 is used in a replay-based manner rather than as a direct numerical substitute for P3: physical offline focus curves and P2 segment-level timing distributions define the baseline replay environment, whereas the measured P3 TS4−TS3 distribution defines only the P3-like actuation tail endpoint for λ_act_ calibration.

In the optical and actuation chain, P3 is implemented as a contrast detection autofocus system composed of a USB industrial camera and a motorized focusing lens, whose focusing actuator is an internal stepper-based mechanism. From the controller perspective, P3 resembles a realistic industrial deployment environment: image acquisition, host-side processing, command generation, interface transfer, and actuator response form a chain that is partly visible but not fully dissectible. P2 does not replicate P3 segment by segment in absolute latency. Instead, it makes the dominant P3 uncertainties scannable while keeping the key mechanisms controllable. P1 provides a near-deterministic reference chain for the low-spread timing limit.

Within this setting, P3 is represented at the control layer by the observable transaction chain segments TS2−TS1, TS3−TS2, and TS4−TS3, together with the unobservable front-end offset Δ_hidden_ = TS1 − t_s_ in T_sa_ = Δ_hidden_ + T_pipe_. Processes that cannot be separately observed on P3, including firmware queues, command batching, status polling, and actuator startup, are therefore not parameterized as independent internal states. Their aggregate control layer effects are carried by the calibrated perturbation coordinates σ_align_ and λ_act_, with σ_align_ representing sample position mismatch and λ_act_ representing actuation chain variability.

All three platforms are analyzed through the same control view: continuous frame input drives focus measure computation and control update, and key event time markers are interpreted under the same logical event definitions. Cross-platform comparison is therefore based on the correspondence among observable temporal segments, controllable actions, and reproducible degradation mechanisms under a common event semantics.

Table 4 summarizes the engineering implementation, approximate capability tier, and analytical role of each platform. P1 and P2 provide the reference and controllable bridge conditions, respectively, whereas P3 is the deployment-side black-box target. In particular, P2 supports replay-based controlled perturbation injection; it is not used as a direct numerical substitute for P3.

### 2.4. Scene Matrix, Strategy Set, and Evaluation Protocol

The evaluated task is contrast detection autofocus under fixed ROI, focus measure, strategy set, and scene set. The core metrics are normalized final quality Q and time to autofocus (TTAF), with Q = F_final_/F_best,off_. Here, F_final_ is the focus score at the final settled position after strategy termination, and F_best,off_ = max(F_off(z)_) is the maximum value of the offline full-sweep focus curve F_off(z)_. The normalization reduces direct scale differences caused by scene texture strength. Together, Q, TTAF, and failure rate represent final quality, time cost, and deployment risk.

TTAF is reported using p50 (the median) for typical time comparison and p99 (the 99th percentile) for tail risk analysis. Aggregated quantities are denoted as Q_mean_ for mean normalized final quality, TTAF_p50_ for median time to autofocus, and TTAF_p99_ for the 99th percentile of time to autofocus. A run is counted as successful when Q > 0.95 and TTAF < 5000 ms. The threshold Q = 0.95 allows approximately 5% normalized quality loss and gives a strict but attainable task tolerance; a substantially higher threshold would approach exact offline optimum hitting, whereas a much lower threshold would be too permissive for precision measurement. The 5000 ms limit represents a seconds-level line pace tolerance for a single autofocus action. Changing these thresholds would change failure rate values, but the main strategy frontier conclusions are determined by the positions of strategies in the Q–TTAF plane. The thresholds therefore affect the later failure-rate comparison but not the frontier geometry.

The test scenes are divided into six categories according to degradation mechanism: high-texture random detail, low-texture/low-contrast broad-peak, pseudo-multipeak caused by periodic repetitive texture, directional texture or single-edge structure, local highlight/specular reflection perturbation, and low-illumination high-noise. For each scene category, four initial conditions are defined: pre-peak far, pre-peak medium, post-peak far, and post-peak medium. The five strategies are denoted as S1–S5, where S1 is continuous move with stop-after-peak detection, S2 is continuous move with stop-after-peak detection plus fixed rollback, S3 is continuous bidirectional bracketing with midpoint selection, S4 is coarse scan + fine scan + early stop, and S5 is hill climbing local search. These settings form the full experimental matrix of 6 scene categories × 4 initial conditions × 5 strategies × 30 repetitions per condition.

Except for the low-illumination scene, all experiments use a unified sampling rate of 80 frames per second (fps) while keeping the ROI, focus measure computation, and strategy decision logic fixed. In the low-illumination scene, only exposure time and analog gain are adjusted to maintain usable images; the autofocus logic, success criterion, and statistical convention are unchanged. Cross-lens validation was conducted on Lens B to test whether the capability-constrained trends and the speed–quality–risk spectrum remain stable under a different optics actuator stack.

The definitions of the six scene categories are listed in Table 5, and the overall evaluation protocol and experimental matrix are summarized in Table 6.

For statistical analysis, variance measures are reported for the 3600-run main experiment on P3 (Lens A). Q is reported with sample standard deviation (SD) and a 95% Student’s t confidence interval (CI) for the mean. TTAF_p50_ and TTAF_p99_ are reported with 95% percentile bootstrap confidence intervals (CIs), using B = 10,000 bootstrap resamples. Failure rate is reported with a 95% Wilson score interval. Strategy-level aggregates use *n* = 720 per strategy and are summarized later in Section 3; scene-stratified cells use *n* = 120 per (scene, strategy) and are reported in Table S5. The P2 perturbation scans reported later in Section 3 are mechanism-level perturbation evidence rather than engineering performance benchmarks, so variance measures are not reported for those scans.

For the P2–P3 degradation pattern comparison, goodness-of-fit (GOF) is computed from degradation-oriented performance components. Let s=1,…,5 index the tested fixed-rule strategies, and let m∈{FR,Q,TTAF} index the three metric channels, where FR*_s_* denotes the failure rate of strategy *s*. The raw performance metrics are first converted into larger-is-worse components:

$$B_{s,\mathrm{FR}}={\mathrm{FR}}_{s},\;\;B_{s,Q}=1-Q_{\mathrm{mean},s},\;\;B_{s,\mathrm{TTAF}}=log\left({\mathrm{TTAF}}_{p99,s}\right).$$

For an injection level a on a given one-factor scan axis, the P2 degradation and the P3 target degradation are measured relative to the same zero-injection P2 baseline:

$$\Delta B_{s,m}^{P2}\left(a\right)=B_{s,m}^{P2}\left(a\right)-B_{s,m}^{P2}\left(0\right),\;\;\Delta B_{s,m}^{P3}=B_{s,m}^{P3}-B_{s,m}^{P2}\left(0\right).$$

For each metric channel *m*, define the scale estimation set as

$$Y_{m}=\{\Delta B_{s,m}^{P2}\left(a\right)|s=1,\ldots ,5,\;a\in \Omega \}\cup \{\Delta B_{s,m}^{P3}|s=1,\ldots ,5\}.$$

where Ω is the injection-level set of that scan axis. The metric-wise scale factor is

$$c_{m}=SD\left(Y_{m}\right).$$

Metric-wise standardization is then applied as

$$x_{s,m}^{P2}\left(a\right)=\frac{\Delta B_{s,m}^{P2}\left(a\right)}{c_{m}+\varepsilon },\;\;x_{s,m}^{P3}=\frac{\Delta B_{s,m}^{P3}}{c_{m}+\varepsilon },$$

where *ε* is a small positive constant used only to avoid division by zero. The standardized components are concatenated into two 15-dimensional vectors:

$$x^{P2}\left(a\right)=\left(x_{1,\mathrm{FR}}^{P2}\left(a\right),\ldots ,x_{5,\mathrm{TTAF}}^{P2}\left(a\right)\right)\in R^{15},\;\;x^{P3}=\left(x_{1,\mathrm{FR}}^{P3},\ldots ,x_{5,\mathrm{TTAF}}^{P3}\right)\in R^{15}.$$

Magnitude proximity is quantified by normalized root-mean-square error (nRMSE) and normalized mean absolute error (nMAE):

$$\mathrm{nRMSE}\left(a\right)=\sqrt{\frac{1}{15}\sum_{j=1}^{15}{\left(x_{j}^{P2}\left(a\right)-x_{j}^{P3}\right)}^{2}},\;\;\mathrm{nMAE}\left(a\right)=\frac{1}{15}\sum_{j=1}^{15}\left|x_{j}^{P2}\left(a\right)-x_{j}^{P3}\right|.$$

The best bridge point on each one-factor scan axis is defined as the injection level with the minimum nRMSE:

$$a^{*}=\underset{a\in \Omega }{argmin}\;\mathrm{nRMSE}\left(a\right),$$

Because $\sigma_{align}$ and $\lambda_{act}$ are scanned separately, the best bridge point is determined independently on each axis rather than as a two-dimensional joint optimum. Cosine similarity, Pearson *r*, Kendall *τ*, and Spearman *ρ* are reported as secondary agreement descriptors for degradation direction, component-shape consistency, and strategy–risk ranking agreement. The mechanism-level correspondence between the one-factor perturbation responses and the P3 phenomena is summarized in Table S3, and complete scan-level GOF values are reported in Table S7.

Overall, the evaluation protocol of this study connects four components: <Z, U, T> capability tiering, TS0–TS4 transaction chain decomposition, P2 replay-based one-factor perturbation along σ_align_ and λ_act_, and GOF comparison with the P3 target pattern using multiple indicators. The role of nRMSE is to select the best bridge point after metric-wise standardization. nMAE, cosine similarity, Pearson *r*, Kendall *τ*, and Spearman *ρ* are used in parallel to describe magnitude proximity, degradation direction agreement, component shape consistency, and strategy–risk ranking agreement.

### 2.5. Replay-Based Controlled Perturbation Procedure

The controlled perturbation procedure on P2 is implemented by replaying strategy logic against measured focus curves and timing distributions under two independent one-factor perturbation scans. The procedure is defined as follows:

1. Obtain the offline full-sweep focus curve F_off_(z) on P2 under the same region of interest (ROI), focus measure, sampling convention. The replay uses a one-step-per-frame convention, meaning that one strategy visible actuator step is associated with one frame interval for time-equivalent calibration. This curve provides the physical focus landscape and F_best,off_ for replay and Q evaluation.
2. Measure the P2 segment-level timing distributions under the unified TS0–TS4 convention. These distributions define the zero-injection baseline replay environment.
3. Use the measured P3 TS4−TS3 distribution only to calibrate the P3-like actuation tail endpoint. No module-level decomposition of the black-box P3 hardware is claimed.
4. Run the σ_align_ scan by adding zero-mean equivalent sample position labeling error to the strategy visible samples on F_off_(z), while holding λ_act_ at the P2 baseline.
5. Run the λ_act_ scan by increasing command-to-actuation variability from the P2 baseline to the P3-like actuation tail endpoint, while holding σ_align_ at baseline.
6. Compute Q, TTAF, failure rate, and goodness-of-fit between each P2 degradation vector and the P3 target degradation vector. The scans are interpreted as mechanism-level perturbation tests, not as unique causal identification of hidden P3 modules.

The σ_align_ scan uses seven uniformly spaced levels, σ_align_ ∈ {0, 1, 2, 3, 4, 5, 6} steps, corresponding to time-equivalent RMS sample position mismatches from 0 to 75.0 ms under the 80 fps one-step-per-frame convention. The λ_act_ scan uses six uniformly spaced levels, λ_act_ ∈ {0, 0.2, 0.4, 0.6, 0.8, 1.0}; λ_act_ = 0 corresponds to the P2 baseline, and λ_act_ = 1.0 corresponds to the P3-like actuation tail endpoint with an equivalent TS4−TS3 p99 of approximately 242.307 ms. The physical values along each axis are linearly mapped between the corresponding baseline and endpoint. The two level sets are independent one-factor scans, not joint perturbation settings. The compact one-factor calibration summary is reported in Table S6.

Table 7 provides a compact reference summary of the key quantities repeatedly used in the analysis; the quantities remain defined at their first substantive occurrence in the text.

## 3. Results

The results are presented in three parts: cross-platform timing distributions and their dominant segments, controlled perturbation results on the bridging platform, and the performance envelope and strategy frontier on the industrial black-box platform.

### 3.1. Cross-Platform End-to-End Latency Distributions and Tail Localization

The three platform types show distinct latency distributions under the unified frame-level transaction chain convention. P1 is nearly deterministic under the present event definitions. P2 adds limited and controlled uncertainty while remaining observable and interpretable. P3 shows a substantial right shift and a pronounced heavy tail within the observable transaction chain. Thus, the platform difference is not only an increase in average latency; it is also a degradation in predictability and tail risk.

For P3, sample-to-actuation latency is not directly juxtaposed with that of P1/P2 because the current interface lacks a sampling reference under the same convention. The P3 timing analysis therefore relies on T_pipe_ and its segment-wise decomposition rather than on side-by-side statistics from inconsistent sampling references.

**Figure 3:**
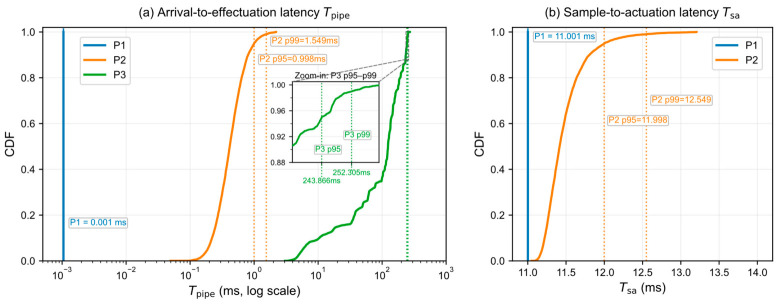
Comparison of end-to-end latency distributions across the three platforms. The figure compares the end-to-end latency distributions of the capability upper-bound platform P1, the bridging platform P2, and the industrial black-box platform P3 under the unified transaction chain definition. Under the current interface, P3 lacks a sampling reference directly comparable with that of P1/P2, so its T_sa_ is not juxtaposed with the first two in the main panel. Panels (**a**) and (**b**) show the arrival-to-effectuation latency T_pipe_ and the sample-to-actuation latency T_sa_, respectively.

The end-to-end latency distributions of the three platforms are shown in Figure 3, and the corresponding quantile statistics are provided in Table S1.

### 3.2. Segment-Wise Decomposition and Structural Differences

Decomposing T_pipe_ into TS2−TS1, TS3−TS2, and TS4−TS3 identifies the main source of tail amplification. P1 remains approximately constant in all three subsegments, and P2 shows only limited expansion. P3 expands in all three subsegments, but the dominant observable tail is concentrated in TS4−TS3. Under the tiering rule in Table 2, P2 falls in the M tier because its observable p99 segment latencies remain within 0.2–5 ms. P3 falls in the L tier because its exposure start reference is unavailable and its TS4−TS3 p99 reaches approximately 242.3 ms, far beyond the 5 ms boundary.

The separation among the three P3 segments is supported by bootstrap confidence intervals and non-parametric tests. With B = 10,000 resamples, TS4−TS3 has a p99 of 242.3 ms (95% CI: [241.5, 243.1]), compared with 20.4 ms for TS2−TS1 (95% CI: [19.9, 21.1]) and 4.0 ms for TS3−TS2 (95% CI: [2.3, 5.7]); the three confidence intervals do not overlap. Kolmogorov−Smirnov and Mann−Whitney U tests with Bonferroni correction (α = 0.017) reject identical distribution assumptions for all three P3 segment pairs (all *p* < 1.0 × 10^−160^). For the key dominance comparison, the Cliff’s δ between TS4−TS3 and TS2−TS1 is 0.84, which is in the large-effect range. As a descriptive ratio between each segment-level marginal p99 and the p99 of observable T_pipe_, TS4−TS3 accounts for 96.0% (95% CI: [95.3%, 96.9%]) of the P3 tail scale. The ratio indicates tail-scale dominance and is not an additive decomposition of the same-run p99 event.

The unobservable P3 front-end offset was evaluated by scanning Δ_hidden_ in T_sa_ = Δ_hidden_ + T_pipe_ over the plausible range [1, 50] ms. The share of TS4−TS3 in the p99 of full T_sa_ is 95.7% at Δ_hidden_ = 1 ms (95% CI: [95.0%, 96.5%]), remains between 89.0% and 94.2% over [5, 20] ms, and is still 80.2% at Δ_hidden_ = 50 ms (95% CI: [79.7%, 80.8%]). The 50% tipping point occurs at Δ_hidden_ ≈ 232 ms, approximately 4.6× beyond the plausible upper bound. The TS4−TS3 tail conclusion is therefore directly observed within T_pipe_ and remains robust when extended to T_sa_ under plausible Δ_hidden_ values.

These results indicate that the main observable tail on P3 is not primarily generated by focus measure computation. It is concentrated between command submission, interface transfer, and the onset of true actuator response. Earlier latency before TS1 may still enter the loop indirectly through sample position mismatch and can interact with post-peak stopping lag, overshoot, and failure rate growth.

**Figure 4:**
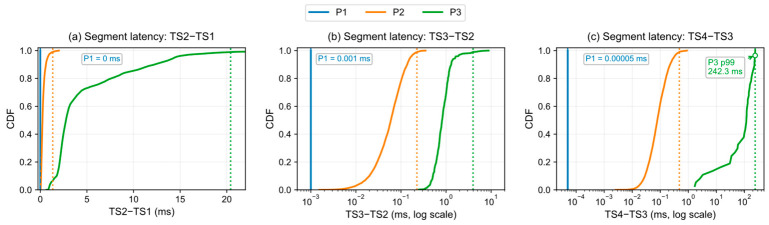
Segment-wise latency decomposition and tail structure across the three platforms. T_pipe_ is decomposed into TS2−TS1, TS3−TS2, and TS4−TS3 to compare the time distributions of processing, control update, and the path from command submission to effective actuation, together with their tail differences, across platforms. Panels (**a**)–(**c**) correspond to TS2−TS1, TS3−TS2, and TS4−TS3, respectively.

The segment-wise decomposition is shown in Figure 4, and the corresponding statistics with confidence intervals are reported in Table S2. The sensitivity scan results for P3 are provided in Table S4.

### 3.3. Single-Strategy Mechanism Illustration and Equivalent Perturbation Abstraction

For the representative strategy of continuous sweep, peak crossing detection, stop, and fixed rollback, the key mechanism is as follows: The true focus peak usually precedes the PEAK hit because the controller observes discrete frame samples and may also apply smoothing, consecutive fall confirmation, or thresholding. A detection lag therefore exists between the true peak and the detected peak.

After the PEAK hit, latency between host-side STOP generation and actual actuator stopping still affects the final landing position. The final position is therefore jointly determined by detection lag and actuation lag. Fixed rollback can compensate average overshoot only when the post-peak stopping offset has a stable statistical center. For the industrial black-box platform, the relevant internal factors are abstracted at the control layer into two equivalent perturbations: sample position mismatch and actuation chain variability. Their replay-based controlled perturbation procedure is defined in Section 2.5, and the corresponding perturbation results are reported in Section 3.4. The compensation limit of stop-after-peak strategies depends not only on peak detection, but also on post-peak repositioning capability, including response speed, landing accuracy, and the statistical stability of rollback.

**Figure 5:**
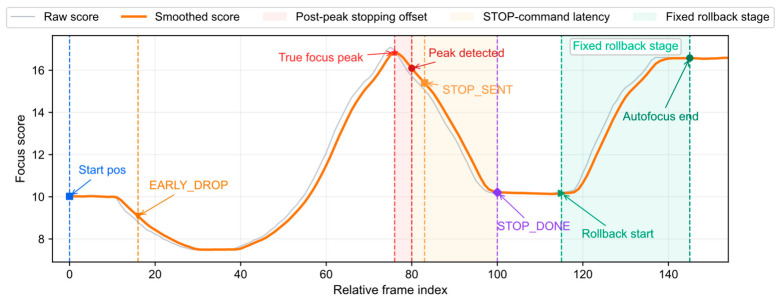
Key-event illustration of stop-after-peak detection and fixed rollback during continuous sweep. The figure shows the true focus peak, the PEAK hit, STOP command issuance, STOP completion confirmation, and fixed rollback completion in one representative autofocus process, together with the timing relationship among the true peak, the detected peak, and the final actuator landing position. The main horizontal axis is the relative frame index, which can be converted into an approximate time scale according to the nominal frame rate.

The key events of the representative single-strategy process are illustrated in Figure 5, and Table 8 summarizes the physical factors, mechanisms, time ranges and typical values, and their equivalent mappings at the control layer.

### 3.4. Controlled Perturbation Results and Mechanism-Level Comparison on the Bridging Platform

**Figure 6:**
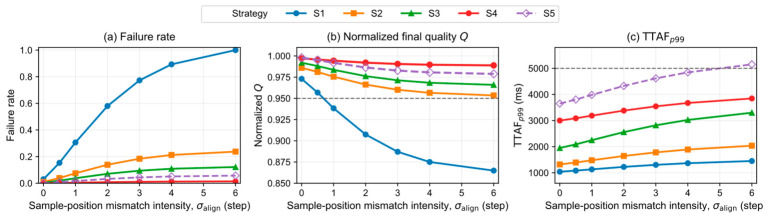
Results of sample position mismatch injection on the bridging platform P2. (**a**) Failure rate versus mismatch intensity σ_align_; (**b**) normalized final quality Q versus σ_align_; (**c**) high-quantile autofocus time TTAF_p99_ versus σ_align_. σ_align_ is expressed in actuator step units and denotes the standard deviation of the equivalent sample position labeling error. Under the nominal 80 fps and one-step-per-frame convention, σ_align_ = 1 corresponds to approximately 12.5 ms and σ_align_ = 6 to approximately 75 ms of time-equivalent RMS sample position mismatch.

**Figure 7:**
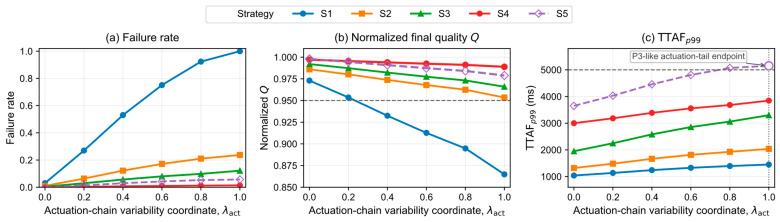
Results of actuation chain variability injection on the bridging platform P2. (**a**) Failure rate versus normalized actuation chain variability coordinate λ_act_; (**b**) normalized final quality Q versus λ_act_; (**c**) high-quantile autofocus time TTAF_p99_ versus λ_act_. λ_act_ is a dimensionless coordinate in [0, 1]. λ_act_ = 0 corresponds to the P2 baseline, whereas λ_act_ = 1 corresponds to the P3-like actuation tail endpoint calibrated from the measured TS4−TS3 distribution, for which the equivalent TS4−TS3 p99 is approximately 242.3 ms. The compact calibration summary is reported in Table S6.

Using the procedure defined in Section 2.5, two one-factor perturbation scans were evaluated on P2. In the σ_align_ scan, λ_act_ was held at the P2 baseline and σ_align_ increased the standard deviation of the equivalent sample position labeling error. In the λ_act_ scan, σ_align_ was held at baseline and λ_act_ increased command-to-actuation variability from the P2 baseline to the P3-like actuation tail endpoint. Figure 6 and Figure 7 show the resulting changes in failure rate, Q, and TTAF_p99_.

The two scans show complementary degradation modes. Increasing σ_align_ reduces final quality and raises failure risk because the focus values used by the strategy are associated with shifted focus axis positions. Increasing λ_act_ broadens the timing uncertainty between command submission and actuation onset, which increases high-quantile completion time and weakens fixed rollback. The scans are therefore interpreted as mechanism-level perturbation evidence for sample position mismatch and actuation chain variability, rather than as module-level causal identification inside P3.

Table 9 summarizes the best bridge GOF results for the two one-factor perturbation scans. The σ_align_ scan reaches its minimum nRMSE at σ_align_ = 6 steps, whereas the λ_act_ scan reaches its minimum nRMSE at λ_act_ = 1.000. In both cases, the best bridge point lies at the high-intensity end of the tested scan range and shows close agreement with the P3 degradation pattern in magnitude, direction, and strategy–risk ranking. Complete scan-level GOF metrics are provided in Table S7.

These results support degradation pattern proximity under controlled equivalent perturbations, not numerical identity between P2 and P3.

### 3.5. Performance Envelope, Strategy Frontier, and Robustness Results on the Industrial Black-Box Platform

On the industrial black-box platform, the unified performance envelope shows no platform-independent global optimum among the tested strategies. Faster strategies generally reduce completion time at the cost of lower quality or higher risk, whereas conservative strategies exchange time cost for more stable imaging. P3 therefore exhibits a continuous speed–quality–risk trade-off spectrum rather than a simple ranking. The strategy frontier here is the empirical frontier of the tested fixed-rule strategies S1–S5, not a universal frontier over all adaptive or hybrid autofocus policies.

S1 and S2 occupy the faster end of the broader S1–S4 speed–quality–risk trade-off band and can be efficient in easier scenes, but they expose more risk under broad-peak, pseudo-multipeak, directional-texture, and low-illumination conditions. Within this band, S3 and S4 form the deployment-preferred subset under the strict success criterion, balancing time, final quality, and failure rate. S5 can show higher upper tail quality in some conditions, but this comes with wider quality dispersion and a heavier time tail; its benefit is therefore not a stable average quality gain. Scene-wise and cross-lens results preserve this trade-off geometry, indicating that the main conclusions are not driven by a single scene or lens stack.

Table 10 summarizes the strategy-level P3 results for the 3600-run Lens A experiment, including mean final quality with sample SD and 95% CI, median and p99 TTAF with 95% bootstrap CIs, and Wilson CIs for the failure rate. Scene-stratified uncertainty measures are reported in Table S5.

**Figure 8:**
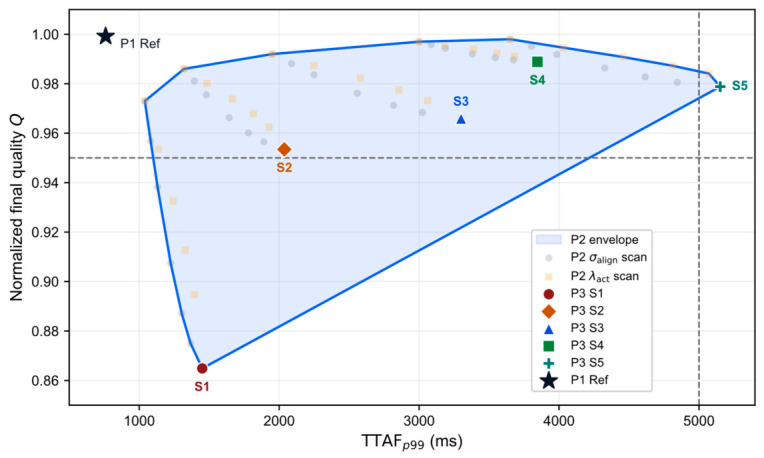
Unified performance envelope across the three platforms. The figure uses normalized quality Q and high-quantile time to autofocus (TTAF) p99 as a common performance plane to show the reachable regions of the capability upper-bound platform P1, the bridging platform P2, and the industrial black-box platform P3. P1 Ref denotes the reference point of the capability upper-bound and does not correspond to any specific strategy index on P3.

**Figure 9:**
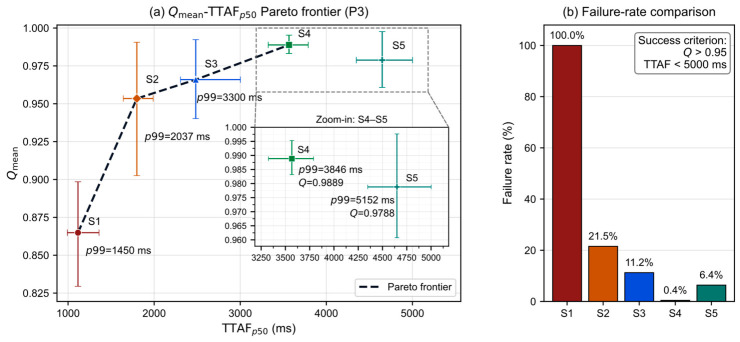
Strategy frontier and failure rate comparison on the industrial black-box platform. (**a**) Pareto plot with TTAF_p50_ on the horizontal axis and Q_mean_ on the vertical axis, characterizing the trade-off position of each strategy between typical autofocus time and average final quality; (**b**) failure rate comparison under the unified success criterion Q > 0.95 and TTAF < 5000 ms, reflecting deployment viability. The plotted frontier is computed only over the tested fixed-rule strategies S1–S5; adaptive, predictive, and online hybrid policies were not part of this empirical comparison. Strategy-level uncertainty estimates are summarized in Table 10.

The unified cross-platform performance envelope is shown in Figure 8, and the strategy frontier together with the failure rate comparison on the industrial black-box platform is shown in Figure 9. Table 10 provides the strategy-level numerical evidence for P3, whereas the scene-wise heatmaps and cross-lens supplementary results are provided in Figures S1 and S2. The strategy-level uncertainty estimates in Table 10 support the speed–quality–risk interpretation derived from Figure 9. S1 is the fastest strategy but does not meet the quality criterion in the tested P3 condition. S2 and S3 improve final quality and reduce the failure rate at the cost of longer TTAF. S4 provides the most stable high-quality result with the lowest failure rate, whereas S5 increases TTAF and failure risk relative to S4 while not improving mean final quality. Therefore, within the tested fixed-rule strategy layer on P3, S1–S4 form the main engineering trade-off band, and S5 does not establish a stable frontier advantage.

## 4. Discussion

The discussion below defines the scope of these variables, the role of P2 as a bridge, the transfer boundary of the conclusions, and the implications for platform evolution and strategy selection.

### 4.1. Interpretive Scope and Non-Uniqueness of the Two Equivalent Variables

σ_align_ and λ_act_ are not one-to-one physical ground truths for the hidden processes in the industrial black-box platform. They are equivalent variables at the control layer describing observable degradation mechanisms. σ_align_ represents the standard deviation of equivalent sample position labeling error along the focus axis and is interpreted as a time-equivalent mismatch only under the one-step-per-frame convention. λ_act_ is a normalized coordinate for actuation chain variability, with its corresponding TS4−TS3 p99 values reported in Table S6; full segment-wise statistics are reported in Table S2.

These variables are not intended to identify a unique internal module in the black-box platform. They provide a scannable and reproducible mechanism coordinate system linked to control outcomes. They remain informative for post-peak stopping lag, overshoot, failure rate, and tail-time behavior in continuous frame-driven CDAF, but should not be used as unique hardware-level truth for finer device diagnostics. The exposure-to-frame-ready segment that is not directly observable on P3 enters the analysis only through the Δ_hidden_ sensitivity scan; the dominance conclusion is robust within the plausible range (Section 3.2 and Table S4) but should not be extrapolated under an assumption that this unobservable segment is spatially decoupled from TS4−TS3.

### 4.2. Why the Bridging Platform Is Necessary

Without P2, the analysis would be split between two unsuitable endpoints. P1 is too idealized to expose the degradation patterns of the industrial black-box platform, whereas P3 is too opaque to separate and scan the relevant factors. P2 fills this gap: it preserves the control logic and partial temporal observability while allowing controlled uncertainty injection [10,14].

Accordingly, P2 is used to provide mechanism-level perturbation evidence, not numerical substitution. It converts the observational question on P3 into a controlled test on P2: whether sample position mismatch and actuation chain variability can reproduce the main observable degradation phenomena. The GOF analysis quantifies this bridge by testing whether perturbations along the two control layer coordinates move the degradation vector toward the P3 target pattern. GOF should therefore be interpreted as degradation pattern proximity, not as numerical identity between P2 and P3 or a complete reconstruction of P3 by a single perturbation axis.

### 4.3. Transfer Boundaries of the Conclusions

The transferable content of this study is the relation between platform capability and performance behavior, not absolute numerical values detached from the capability context. P1, P2, and P3 represent an upper-bound platform, a bridging intermediate layer, and an industrial black-box deployment condition. A strategy advantage observed on P1 or P2 can transfer only when the target platform provides comparable observability, controllability, and timing determinism.

The long-tail and risk structure observed on P3 should therefore not be generalized mechanically to every industrial platform. Higher event time visibility, stronger actuator feedback, or more deterministic interfaces may change the strategy frontier and its speed–quality–risk geometry [9,11,14]. The framework provides capability-conditioned interpretation rather than a fixed platform-independent ranking.

### 4.4. Engineering Implications for Platform Capability Evolution

Deployed CDAF is unlikely to improve substantially through faster host-side computation alone. As Section 3.2 shows, the dominant observable tail on P3 occurs after the decision, so the main engineering priorities are actuation path stabilization, exposure of key transaction chain timestamps, and real-time position feedback. Under the tiering rule in Section 2.1 and Table 2, these priorities correspond to the U, T, and Z dimensions, respectively [9,15,16].

Along the *Z*-axis, the current platform lacks real-time position readout during motion and is therefore in the L tier. Actuator-aligned position sensing—such as optical encoders for stepper actuators, Hall sensors for voice coil motors (VCMs), or capacitive sensors for piezoelectric stages—can bring position feedback p99 latency below the 5 ms boundary (M tier) or, in fast implementations, below 0.2 ms (H tier). Such observability enables midpoint verification, online overshoot correction, and model-based delay compensation; without it, the controller can only use position information after motion completion.

Along the U-axis, P3 is limited by the post-decision–actuation segment TS4−TS3, whose p99 is approximately 242.3 ms. The upgrade path includes both actuator-level and link-level changes: faster actuators such as VCMs, piezoelectric stages, liquid lenses, or ultra-thin back-focus mechanisms reduce physical response time, while direct low-level control or bounded-queue interfaces reduce command chain uncertainty. Together, these changes aim to move command-to-effective-actuation p99 below 5 ms and, for short-stroke systems, potentially below 0.2 ms.

Along the T-axis, P3 lacks a direct timestamp for exposure start TS0 and exposes actuation onset only indirectly through busy/status information. The corresponding upgrade path is to open the transaction chain markers: hardware exposure start timestamps, explicit command-sent marker and actuation onset acknowledgments, hardware triggers or Precision Time Protocol (PTP)-synchronized interfaces, and more deterministic driver response. These measures improve timing observability and determinism without necessarily changing the actuator itself and can move the relevant p99 uncertainties from the L tier toward the M tier.

Table 11 summarizes the improvement pathways, target axes, and T_pipe_ segments most likely to benefit. In L-tier systems, weak timing observability and actuation controllability narrow the strategy design space; delay-aware prediction, online compensation, event-driven policies, and adaptive or hybrid strategies become stable only after one or more of Z, U, and T is lifted to M or H.

### 4.5. Deployment Decision Procedure Under Platform Capability Constraints

The framework, transaction chain, and strategy frontier results can be organized into a three-step deployment screening procedure that maps a target camera–lens–host configuration to a candidate strategy band and a platform improvement priority. The procedure uses the tiering rule in Table 2, the T_pipe_ decomposition in Section 3.2, the success criterion in Section 2.4, and the strategy frontier results in Figure 9 and Table 10. It does not replace target system validation of Q, TTAF, and failure risk; instead, it identifies which strategy subset and improvement axis should be evaluated first.

Table 11 serves as the platform improvement catalog, whereas Table 12 is the deployment-side decision matrix; the latter points back to Table 11 only when a capability upgrade is required.

Step 1—capability tiering. Determine Δz_min_ and T_ref_ for the target system, then measure the p99 uncertainty of position feedback, command-to-effect latency, and key event timing. Assign H, M, or L to Z, U, and T using Table 2. The resulting triplet <Z, U, T> locates the target platform in the capability space.

Step 2—bottleneck localization. Using the event semantics in Section 2.2, measure the p99 of TS2−TS1, TS3−TS2, and TS4−TS3 on the target system, and identify the dominant segment. For the P3 system in this study, TS4−TS3 is dominant, supported by non-overlapping bootstrap 95% CIs and by Kolmogorov–Smirnov and Mann–Whitney U tests at α = 0.017 (Section 3.2). A target system in the same capability tier may show a similar post-decision bottleneck, but Step 3 should use the dominant segment measured on that system.

Step 3—strategy band and improvement priority. Use the capability triplet from Step 1, the dominant segment from Step 2, and the task tolerance expressed by Section 2.4 success criterion as inputs to Table 12. The output is a candidate strategy band within the tested S1–S5 set and a corresponding Z-, U-, or T-axis improvement priority. The measured P3 system listed in Table 4 consists of a USB industrial camera coupled to a command-driven motorized focusing lens with an internal stepper-based focusing actuator. Applied to this system, the inputs from Steps 1 and 2 place the platform in the <L, L, L> cell of Table 12, where TS4−TS3 is the dominant segment. On P3, this assignment follows from the absence of real-time position readout and a direct exposure start timestamp, together with the measured TS4−TS3 p99 of approximately 242.3 ms. In this cell, the strategy-level results in Table 10 support the interpretation that S1–S4 form a descriptive speed–quality–risk trade-off band, whereas S3–S4 are the deployment-preferred subset under the strict success criterion. S2 may be selected when faster completion is prioritized and moderate failure risk is acceptable, while S1 is retained only as a speed-oriented baseline. U-axis improvement remains the first priority, followed by T-axis improvement.

The procedure connects capability tiering, bottleneck localization, and strategy frontier geometry. It does not transfer the Q, TTAF, or TS4−TS3 values measured on P3 to other platforms. Consistent with Section 4.7, transfer is achieved by capability-tier matching and directional recommendation, not by cross-platform numerical prediction.

### 4.6. Implications for Strategy Design

For stop-after-peak and compensation-based strategies, engineering usefulness depends not only on peak detection sensitivity, but also on whether the system can reposition quickly, accurately, and consistently after crossing the peak. When post-peak repositioning is weak, strategy design should avoid aggressive stopping and fixed rollback assumptions and should instead control delay sensitivity and tail risk. Strategy selection is therefore an adaptation to platform capability, not an algorithm preference detached from the platform.

The P3 results show that deployment should not seek a single globally optimal strategy. S1–S4 form the main descriptive speed–quality–risk trade-off band, whereas the advantage of S5 is limited to condition-dependent upper-quantile quality gains and does not form a stable frontier position. Under the strict success criterion in Section 2.4, S3–S4 are the deployment-preferred subset for the P3 condition; S2 may be considered when faster completion is prioritized and moderate failure risk is acceptable, and S1 should be treated as a speed-oriented baseline rather than a successful deployment choice. The ceiling of compensation-based strategies is then set by how far the Z, U, and T-axes can be lifted by the platform improvements summarized in Table 11.

### 4.7. Study Limitations

Several limitations remain. First, the front-end portion of the P3 chain is not directly observable, so the analysis cannot resolve every internal black-box module. Second, the equivalent variables used in P2 are mechanism-level abstractions rather than unique physical truths; they support mechanism-level interpretation, but not unique module-level causal diagnosis or replacement of hardware-specific testing.

The strategy set is also limited. The experiments evaluate five fixed-rule, frame-driven strategies and do not test adaptive, predictive, learning-based, or online hybrid policies. This does not imply that such policies are ineffective; rather, their reproducible evaluation requires aligned timing markers, identifiable delay models, and exposed actuator state or position feedback, which the current P3 interface does not provide. The S1–S4 trade-off band and the absence of a stable S5 frontier advantage should therefore be read as conclusions for the tested S1–S5 layer under the P3 capability condition.

External validation is further limited to one actuator–interface type: a stepper-driven motorized focusing lens with a USB industrial camera. The two lens implementations provide within-family optical and sample-level robustness, but not validation across actuator technologies or communication interfaces. The absolute P3 values should therefore not be generalized to voice coil, piezoelectric, or liquid lens systems, or to GigE Vision, PTP-synchronized, or CoaXPress platforms. What may transfer is the capability-conditioned form of the conclusions: platforms in a similar <Z, U, T> cell may show similar trade-off geometry, whereas platforms moved upward in the capability grid may reorganize the frontier.

## 5. Conclusions

CDAF evaluation is recast as capability-conditioned trade-off analysis rather than platform-independent algorithm ranking. P1, P2, and P3 are treated as anchor points in a shared <Z, U, T> capability space rather than as isolated devices. Differences in completion time, final quality, failure rate, and strategy ordering are interpreted through platform capability conditions, not as intrinsic properties of the tested strategies alone.

The analysis rests on three instruments: the <Z, U, T> tiering rule anchored by Δz_min_ and T_ref_, the platform-independent transaction chain TS0–TS4 with T_pipe_ and T_sa_, and the calibrated perturbation coordinates σ_align_ and λ_act_. These instruments make the observability, controllability, and timing determinism assumptions behind CDAF strategies explicit and comparable.

In the measured P3 condition, the dominant observable tail on P3 is localized in TS4−TS3. The P2 one-factor perturbation scans along σ_align_ and λ_act_ reproduce the P3-like degradation pattern at their best bridge points, and the P3 strategy-level results support an S1–S4 speed–quality–risk trade-off band rather than a stable S5 frontier advantage.

For deployment screening, the results are organized into a platform improvement catalog and a deployment-side decision matrix. Together they support preliminary capability tiering, dominant segment localization, strategy band selection, and platform improvement prioritization. Final deployment still requires verifying Q, TTAF, and failure risk on the target hardware.

This use is bounded. The framework can be applied to CDAF systems in the same or adjacent capability cells, but the specific frontier geometry, strategy band boundary, and numerical thresholds must be verified on the target hardware. The study does not claim transfer to non-focus frame-driven control tasks with different control targets or actuation semantics.

## Figures and Tables

**Table 1:** CDAF method families, platform prerequisites, typical failure conditions, and representative references.

Method Family	Case	Implicit Platform Prerequisite	Typical Failure Condition	Representative References
Continuous sweep with stop-after-peak detection	S1	Sampling–control latency is small and weakly varying; peak crossing can trigger stopping in time	Delayed stopping amplifies overshoot; failure rate rises under heavy-tailed latency	[6,9]
Stop-after-peak detection with fixed rollback	S2	Mean overshoot is relatively stable and can be approximated by a constant rollback	Only the mean offset can be compensated; long tails and strong fluctuations remain	[6]
Bidirectional bracketing with midpoint selection	S3	Samples from both sides are available, and position readout or direction switching remains sufficiently controllable	Benefit decreases when position observability is poor or direction switching is unstable	[1]
Coarse-to-fine two-stage search	S4	The search space is relatively stable, and the switching cost between coarse and fine scan is controllable	Under large tail fluctuations, the gain of multi-stage switching can be offset by uncertainty	[1,9]
Local hill climbing	S5	Local unimodality is reasonably good, and sampling feedback and control update can remain closed-loop	Low texture, intensified sample position mismatch, or pronounced actuation lag can cause oscillation and local stagnation	[5]
Predictive/event-driven extensions	—	Event time marking, triggering, and synchronization interfaces are more open, such that delay models are identifiable or events can be aligned	Stable implementation is difficult on black-box platforms because of missing interfaces, model drift, or heavy-tailed fluctuations	[14]

**Table 2:** Operational quantitative tiering of the platform capability triplet <Z, U, T> based on the task scale displacement Δz_min_ and reference time T_ref_.

Dimension	H	M	L
Z: position observability	Real-time position during motion; τ_Z,p99_ ≤ 0.2 ms; readout resolves Δz_min_ scale.	Position or status available but τ_Z,p99_ = 0.2–5 ms.	No real-time position; post-motion only or busy/status only; or τ_Z,p99_ > 5 ms.
U: actuation controllability	Direct low-level control; command-to-effect τ_U,p99_ ≤ 0.2 ms.	CPU/driver/communication control with bounded delay; τ_U,p99_ = 0.2–5 ms.	Opaque threaded/interface/device queue or heavy tail; τ_U,p99_ > 5 ms or onset unavailable.
T: timing observability and determinism	TS0/exposure, exposure duration, frame arrival, FM completion, control commit, actuation onset observable; τ_T,p99_ ≤ 0.2 ms.	Core events available, but CPU scheduling/DDR/OS introduces τ_T,p99_ = 0.2–5 ms.	Exposure start or key marker unavailable, or any critical timing segment p99 > 5 ms.

**Table 3:** Observation mapping of the logical events TS0–TS4 across the three platform types. The table lists the corresponding event time markers or observable events for each unified logical event on each platform to support the cross-platform definition of the transaction chain.

Logical Event	P1 Tier (FPGA Hard Real-Time/Direct Control)	P2 Tier (CPU + DDR)	P3 Tier (Industrial Camera USB/GigE + Command-Driven Lens)
TS0	Exposure start (available through FPGA/sensor hardware synchronization)	Exposure start (sensor-side/driver event time marker or recorded by the acquisition thread)	Exposure start not directly observable; usually only frame-level time markers or indirect estimates are available
TS1	Frame arrival at the computable domain: FPGA RX/image signal processor input arrival (hardware event time marking available)	Frame arrival at the computable domain: moment when DMA reception completes/buffer becomes readable on the processor	Frame arrival at the host-side computable domain: SDK callback or frame grab completion (including USB/GigE, driver, copy, and scheduling)
TS2	FM ready: focus measure computation completed	FM ready: focus measure computation completed (possibly including DDR/buffer access)	FM ready: ROI/FM computation completed on the host
TS3	Transaction submission (commit): the strategy outputs the action and submits it to the actuation chain	Transaction submission (commit): the strategy outputs the action and submits it to the actuation chain	Transaction submission (commit): the host generates, queues, and prepares the command for transmission
TS3.5 (optional)	First effective pulse scheduled/output (often approx. TS4)	Command sent to the driver/peripheral interface	Command-sent marker: command leaves the host (but the device may not yet have started execution)
TS4	Actuation takes effect (apply): motor/lens begins to move	Actuation takes effect (apply): driver starts output/motor begins to move	Actuation takes effect (apply): lens/device actually starts moving (usually inferred indirectly via busy/status confirmation)

**Table 4:** Platform roles and engineering implementation.

Platform	Engineering Implementation	Approximate Capability Tier	Analytical Role in This Study
P1	FPGA-based hard-real-time low-level direct-control reference chain	<H, H, H>	Capability upper-bound reference for low-dispersion timing and high-observability event marking
P2	CPU + DDR bridging replay environment with observable timing and repeatable strategy logic	<M, M, M>	Provides the offline full-sweep focus curve, zero-injection baseline timing distributions, and the replay environment for one-factor perturbation injection
P3	USB industrial camera + command-driven motorized focusing lens with an internal stepper-based focusing actuator	<L, L, L>	Industrial black-box target for deployment-side timing evidence, strategy frontier analysis, and bottleneck localization

**Table 5:** Definitions and construction principles of the six scene categories.

Scene Category	Degradation Mechanism, Construction Principle, and Typical Examples	Primary Challenge
High-texture random-detail scene	High-frequency random detail is used to form a stable single-peak response; used as the baseline scene. Typical examples: printed text paper, fine woven fabric, random granular surfaces.	Baseline condition for observing the speed–quality trade-off under information-rich input.
Low-texture/low-contrast broad peak scene	Texture strength and contrast are reduced to form a broad peak or plateau-like peak. Typical examples: lightly textured paper, weak-contrast planar materials.	Broad peaks and weak gradients increase hesitation in stopping and raise time and failure risk.
Pseudo-multipeak scene from periodic repetitive texture	Periodic repetitive texture is introduced to create false peaks and shoulder peaks. Typical examples: regular stripes, periodic grids, repeated patterns.	False peaks and shoulder peaks increase the risk of mistaken stopping, local stagnation, or rank reordering among strategies.
Directional texture/single-edge scene	Single-sided edges or strongly directional texture are constructed to accentuate ROI geometric sensitivity. Typical examples: one-sided edge rulers, brushed textures, tilted-edge targets.	Stronger ROI geometric sensitivity can amplify directional bias and local criterion instability.
Local highlight/specular reflection perturbation scene	Local bright spots or specular reflections are used to perturb the local response and shift the peak position. Typical examples: reflective metal parts, local reflective patches, specular highlight regions.	Local response perturbations can shift the apparent peak and induce mistaken stopping or quality loss.
Low-illumination high-noise scene	Illumination is reduced and exposure/gain are adjusted accordingly to amplify noise and frame-to-frame fluctuation. Typical examples: low-light paper or textured targets captured under elevated gain.	Noise and frame-to-frame fluctuation increase jointly, often worsening quality, tail time, and failure risk.

**Table 6:** Overview of the evaluation protocol and experimental matrix.

Dimension	Setting	Notes
Platform levels	P1 capability upper-bound/P2 bridging platform/P3 industrial black-box platform	Provide upper-bound reference, mechanism validation, and target deployment evidence, respectively; P3 is the industrial target platform that presents black-box behavior to the controller
Scene categories	6 degradation mechanism scene categories	High-texture random detail, low-texture/low-contrast broad peak, pseudo-multipeak from periodic repetitive texture, directional texture/single-edge structure, local highlight/specular reflection perturbation, and low-illumination high-noise (see Table 5 for definitions, construction principles, and examples)
Initial conditions	4 types	Pre-peak far, pre-peak medium, post-peak far, post-peak medium
Strategy set	5 types	S1 continuous move with stop-after-peak detection; S2 continuous move with stop-after-peak detection and fixed rollback; S3 bidirectional bracketing with midpoint selection; S4 coarse sweep + fine sweep + early stopping; S5 hill climbing
Repetitions	30 runs per combination	Each scene × initial condition × strategy combination is repeated 30 times
Experimental scale	3600 autofocus runs	6 scenes × 4 initial conditions × 5 strategies × 30 repetitions; 720 runs per strategy
Sampling rate	80 fps	Fixed for the main experiment; only exposure/gain are adjusted in the low-illumination high-noise scene, without changing the autofocus logic
Unified settings	Unified ROI/FM/success criterion	Q, TTAF (p50/p99) and failure rate are reported under the same statistical convention
Cross-lens validation	Completed	Independent full experimental validation on both Lens A and Lens B; used to verify trend stability rather than exact numerical agreement

**Table 7:** Reference summary of key quantities repeatedly used in the analysis.

Quantity	Meaning in This Paper	Main Use
Δz_min_	Minimum effective focus displacement.	Task scale anchor for capability tiering.
T_ref_	Reference time required for the actuator to travel Δz_min_.	Sets task scale timing boundaries.
<Z, U, T>	Capability triplet for position observability, actuation controllability, and timing observability and determinism.	Locates platform capability tiers.
τ_X,p99_	p99 uncertainty of feedback, command-to-effect behavior, or event timing along X ∈ {Z, U, T}.	Assigns H/M/L tiers.
t_s_	Sampling instant, t_s_ = TS0 + T_exp_/2.	Defines sample-to-actuation timing where TS0 is observable.
T_pipe_	Arrival-to-effectuation latency, T_pipe_ = TS4 − TS1.	Main observable cross-platform timing metric.
T_sa_	Sample-to-actuation latency, T_sa_ = TS4 − t_s_.	Physical sample actuation mismatch metric where a sampling reference is available.
Δ_hidden_	Unobservable front-end offset on P3, Δ_hidden_ = TS1 − t_s_.	Used in the observability boundary sensitivity analysis.
Q	Normalized final quality, Q = F_final_/F_best,off_.	Final focusing quality metric.
TTAF	Time to autofocus.	Speed metric, reported using p50 and p99.
σ_align_	Standard deviation of equivalent sample position labeling error along the focus axis.	P2 replay coordinate for sample position mismatch.
λ_act_	Normalized actuation chain variability coordinate in [0, 1].	P2 replay coordinate for command-to-actuation variability.
GOF metrics	nRMSE, nMAE, cosine similarity, Pearson *r*, Kendall *τ*, and Spearman *ρ*.	Compare P2 perturbation scans with the P3 degradation pattern.

**Table 8:** Physical factors, mechanisms, time ranges and typical values and their equivalent mappings at the control layer. The listed time values describe observable or equivalent time ranges. For P3 internal actuation and firmware factors that cannot be directly decomposed, no module-level timing is claimed; these factors are jointly projected to TS4−TS3 and carried by the λ_act_ endpoint calibration in Table S6.

Underlying Physical Factor	Main Mechanism	Time Range and Typical Value	Control-Visible Equivalent Effect	Main Mapped Variable
Exposure integration and frame phase	The actuator may continue moving during exposure, so the focus score corresponds to an optical state integrated over the exposure window; frame-level marking may therefore shift the sample position.	At 80 fps, one frame is 12.5 ms; if the phase is unknown, a half-frame scale is about 6.25 ms. In Table S6, σ_align_ = 1 step corresponds to a 12.5 ms RMS equivalent mismatch.	The focus sample is labeled at a shifted focus axis position, which can bias peak crossing detection.	σ_align_
Sensor readout, buffering, transport, and SDK callback front end	After exposure ends, the frame passes through an encapsulated chain before it enters the computable domain. On P3, TS0 is not directly observable, so this part enters the analysis as a hidden front-end offset.	P3 uses Δ_hidden_ = 1–50 ms as the sensitivity range; the typical discussion range is 5–20 ms. This effect enters the analysis only through the sensitivity scan over Δ_hidden_.	The physical position represented by a sample lags behind the controller processing time, forming sample position mismatch.	σ_align_
Host-side ROI/FM computation and memory access	After frame arrival, ROI access, focus measure computation, and memory access form TS2−TS1.	P2: mean 0.302 ms, p99 1.336 ms; P3: mean 4.816 ms, p99 20.415 ms.	The decision basis is delayed relative to the moving optical state; this mainly appears as sample position mismatch and is not the main P3 tail source.	σ_align_
Control update and command preparation	After focus measure computation, strategy update, action generation, and command queuing form TS3−TS2.	P2: mean 0.070 ms, p99 0.226 ms; P3: mean 0.947 ms, p99 3.975 ms.	This introduces lightweight decision–actuation mismatch and may contribute to delayed stopping, but less than TS4−TS3.	σ_align_; weak λ_act_
Host-to-lens or lower-level command transfer	STOP, rollback, or move commands are submitted by the host and pass through interface transfer, protocol handling, and device-side reception, entering TS4−TS3.	P2 TS4−TS3: mean 0.109 ms, p99 0.479 ms; P3 TS4−TS3: mean 112.867 ms, p99 242.307 ms.	Long-tailed command-to-effect time makes the spatial projection of stopping and rollback unstable.	λ_act_
Driver buffering, firmware queues, and lower-level batching	The device side may buffer commands, poll status, batch transactions, or queue execution; the controller sees only the combined result between command submission and effectuation.	Not decomposed module by module inside P3; calibrated as a unified TS4−TS3 baseline-to-endpoint distribution.	The same STOP command may take effect at different physical positions across repetitions, amplifying tail risk.	λ_act_
Actuator startup dead zone and motion establishment	After a command is accepted, electrical response, micro-step establishment, and mechanical clearance must be overcome before visible motion begins.	No separate module-level time is assigned; this is included in TS4−TS3. The P3-like actuation tail endpoint p99 is 242.307 ms.	The effective delay of stop or reverse commands lowers the ability of fixed rollback to compress the tail.	λ_act_
Mechanical hysteresis, micro-nonlinearity, backlash, and settling	The same command may not land at exactly the same final position, especially after peak crossing and fixed rollback.	No independent event timestamp is available; this effect is carried by the λ_act_ endpoint distribution at the control level.	The final landing position distribution widens, Q decreases, and TTAF tail and failure rate increase.	Primarily λ_act_

**Table 9:** Best bridge goodness-of-fit summary for the two one-factor perturbation scans. nRMSE and nMAE quantify magnitude proximity; cosine similarity and Pearson *r* quantify directional and component shape consistency; rank agreement reports Kendall *τ* and Spearman *ρ*. The high-intensity endpoint in each scan provides the nearest P3-like degradation pattern within the tested scan range.

Perturbation Axis (Mechanism)	Best Bridge Point	nRMSE	nMAE	Cosine	Pearson *r*	Rank Agreement
σ_align_ (sample position mismatch)	6 steps	0.0256	0.0118	0.9999	0.9998	Kendall *τ* = 1.000; Spearman *ρ* = 1.000
λ_act_ (actuation chain variability)	1.000	0.0244	0.0113	0.9999	0.9998	Kendall *τ* = 1.000; Spearman *ρ* = 1.000

**Table 10:** Strategy-level performance summary on P3 (Lens A) for the 3600-run experiment. Each strategy is evaluated with *n* = 720 runs. Q_mean_ is reported with sample SD and a 95% Student’s t-interval; TTAF_p50_ and TTAF_p99_ use 95% percentile bootstrap CIs (B = 10,000); failure rate uses a 95% Wilson score CI. Failure is defined as Q ≤ 0.95 or TTAF ≥ 5000 ms.

Strategy	Q_mean_ (SD)	Q 95% CI	TTAF_p50_ 95% CI (ms)	TTAF_p99_ 95% CI (ms)	Failure Rate [95% Wilson CI]
S1	0.8649 (0.0275)	[0.8629, 0.8669]	[1102.0, 1129.3]	[1404.9, 1489.2]	100.00% [99.47, 100.00]
S2	0.9535 (0.0338)	[0.9510, 0.9559]	[1786.4, 1813.0]	[2023.1, 2066.3]	21.53% [18.68, 24.68]
S3	0.9659 (0.0260)	[0.9640, 0.9678]	[2463.2, 2509.8]	[3186.6, 3366.3]	11.25% [9.14, 13.77]
S4	0.9889 (0.0069)	[0.9884, 0.9894]	[3548.3, 3583.8]	[3829.6, 3888.1]	0.42% [0.14, 1.22]
S5	0.9788 (0.0145)	[0.9778, 0.9799]	[4625.8, 4672.5]	[5095.5, 5228.7]	6.39% [4.82, 8.42]

**Table 11:** Improvement pathways for platform capability evolution organized by the <Z, U, T> axes, with target tiers (with reference to Table 2) and the T_pipe_ segments expected to be most affected. The symbol “→” denotes a capability-tier upgrade, and “↓” denotes a reduction in the corresponding latency segment.

Axis	Engineering Pathway	Specific Means	Target Tier	Most Affected Segment
Z position observability	Integrate real-time position feedback aligned with the actuator	Optical encoder for stepper actuators; Hall-based sensor for VCM; capacitive sensor for piezoelectric stages; in-motion readout at Δz_min_ resolution	L → M or H	Enables post-peak verification; no direct T_pipe_ shortening but supports U and T upgrades
U actuation controllability	High-response actuator + direct low-level control	VCM/piezoelectric/liquid lens/ultra-thin back-focus actuator; replace opaque multi-stage command chain by voltage/current/pulse/frequency control, or adopt interfaces with predictable bounded queuing	L → M or H	TS4−TS3 ↓; TS3−TS2 ↓
T timing observability and determinism	Open key event markers along the transaction chain	Hardware-level TS0 (exposure start) timestamps; explicit command-sent marker and actuation onset acknowledgment; hardware triggers/PTP-synchronized interfaces; deterministic OS-level driver response	L → M	Makes TS0/exposure start and Δ_hidden_ observable; TS3−TS2, TS4−TS3 ↓

**Table 12:** Deployment decision matrix. “Strategy band” denotes a candidate or preferred subset within the tested fixed-rule strategies S1–S5 under the capability condition, dominant transaction chain segment, and task tolerance defined in Section 2.4. “Trade-off band” denotes the observed speed–quality–risk spectrum within the tested strategy set, whereas “deployment preference” denotes the subset favored after applying the success criterion and risk tolerance. “Improvement pathway” cross-references Table 11 rows labeled by the capability axis (Z/U/T). Entries for platform tiers not directly represented by the paper’s P1–P3 anchors should be read as conservative framework extrapolations rather than directly measured results.

<Z, U, T> Tier	TS2−TS1 Dominant	TS3−TS2 Dominant	TS4−TS3 Dominant
L–L–L (P3-like)	Front-end processing bottleneck. Strategy band: conservative S3–S4; improvement: T and/or host pipeline.	Control update bottleneck. Strategy band: S3–S4; improvement: T + U link level.	P3-like black-box case. Trade-off band: S1–S4. Deployment preference: S3–S4; S2 only for speed-critical tasks with moderate risk tolerance. S5 not recommended. Improvement: U first, T second.
L/M mixed (e.g., T at M, U at L)	Strategy band: S2–S4; improvement: lift the limiting T-related axis.	Strategy band: S2–S4; improvement: T + U link level.	Strategy band: S1–S4, with S2/S4 preferred; improvement: U actuator.
M–M–M (P2-like)	Strategy band: broad S1–S4; delay-aware variants become feasible.	Strategy band: broad S1–S4; delay-aware variants feasible.	Strategy band: broad S1–S4; S5 may be used under lenient tolerance; improvement: U.
H–H–H (P1-like)	Classical strategies feasible; predictive, event-driven, and adaptive extensions become stable.	Classical strategies feasible; predictive, event-driven, and adaptive extensions become stable.	TS4−TS3 dominance unlikely; if present, check actuator mismatch or U-axis limitation.

## Data Availability

The source data underlying the main text figures and tables, together with the supplementary figures and tables and the trial-level derived results used for statistical analysis, are provided as a Supplementary Data File accompanying this submission. Complete raw timing logs and device-specific control code are not publicly available because they contain platform-specific industrial control traces and implementation details, but may be made available from the corresponding author on reasonable request.

## Supplementary Materials

The following supporting information can be downloaded at: https://www.mdpi.com/article/10.3390/s26123770/s1, Figure S1: Strategy-stratified heatmaps across six scene categories; Figure S2: Cross-lens comparison of performance frontiers and failure rates; Table S1: End-to-end latency statistics across P1, P2, and P3; Table S2: Segment-level latency statistics across P1, P2, and P3 with standard deviation and bootstrap 95% confidence intervals for the p99 of each segment; Table S3: Correspondence between one-factor perturbation responses on the bridging platform and the main phenomena of the industrial black-box platform; Table S4: Sensitivity of the TS4−TS3 dominance within T_sa_ to the unobservable front-end offset Δ_hidden_ on P3; Table S5: Scene-stratified variance measures (SD and 95% CIs) for Q, TTAF, and failure rate on P3 (Lens A); Table S6: Compact physical calibration summary for the controlled perturbation levels; Table S7: Goodness-of-fit metrics between the P2 one-factor perturbation scans and the P3 degradation pattern.
