# Supplementary material for: Timing Decomposition and Strategy Trade-Offs in Contrast Detection Autofocus Under Platform Capability Constraints
Source: Sensors (Basel). 2026 Jun 12;26(12):3770. doi: 10.3390/s26123770 (PMC13306768; doi:10.3390/s26123770)
Supplement: Supplementary file 1 [file sensors-26-03770-s001.zip › sensors-4258901-supplementary.pdf]

Supplementary Materials

Unless otherwise stated, symbols and abbreviations follow the definitions in the main manuscript; Q_{mean} denotes mean normalized final quality, $TTAF_{p50}$ and $TTAF_{p99}$ denote the median and 99th percentile of time to autofocus, and SD and CI denote standard deviation and confidence interval, respectively.

Supplementary Figures

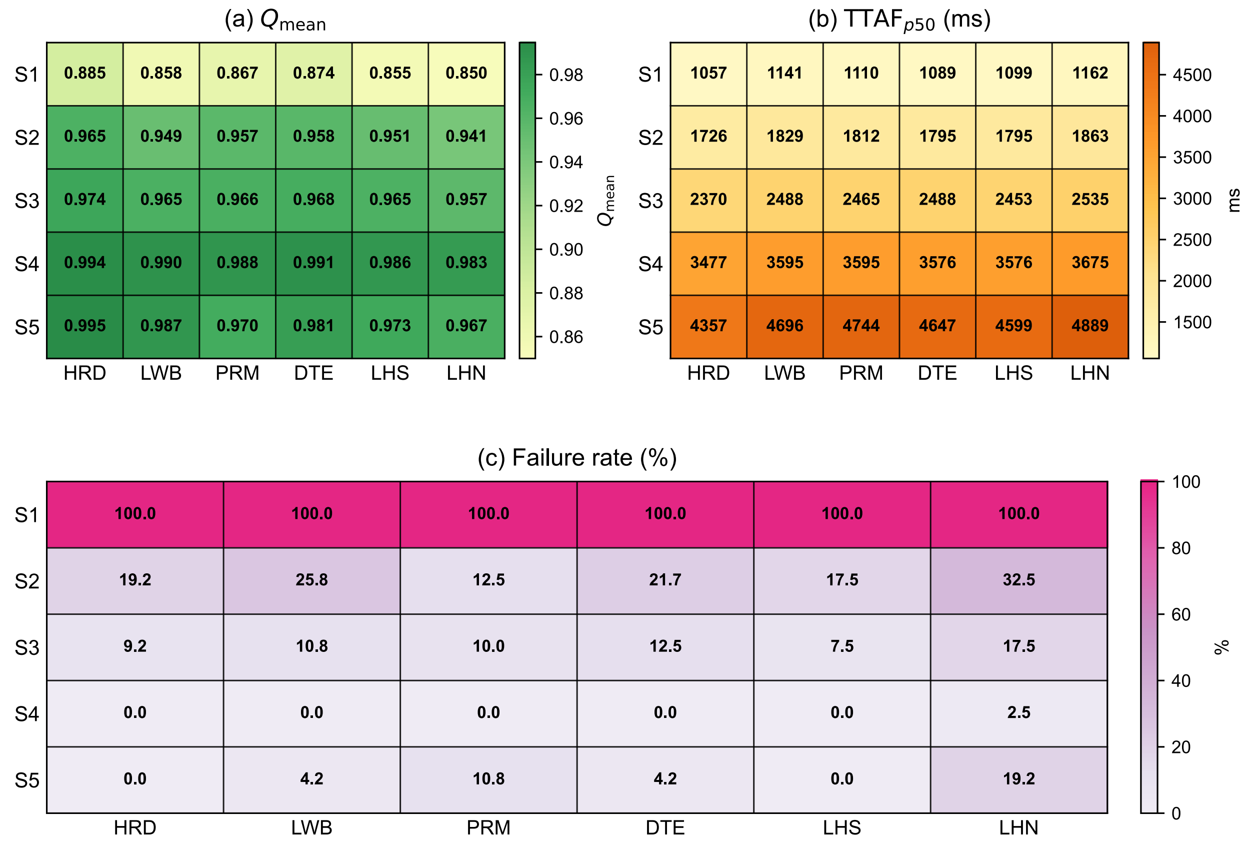

Figure S1. Scene-wise heatmaps of strategy performance across six scene categories. The three rows show Q_{mean}, $TTAF_{p50}$, and failure rate. Scene abbreviations denote high-texture random detail (HRD), low-texture/low-contrast broad peak (LWB), periodic repetitive-texture pseudo-multipeak (PRM), directional texture/single-edge (DTE), local highlight/specular-reflection disturbance (LHS), and low-illumination high-noise (LHN). Per-cell SDs and 95% confidence intervals are provided in Table S5.

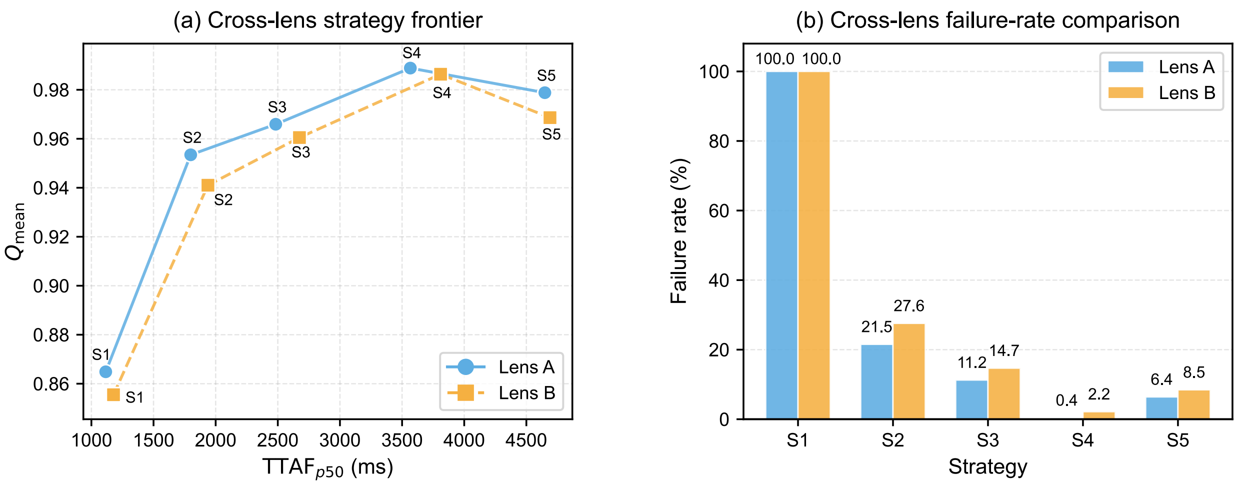

Figure S2. Cross-lens comparison of the strategy frontier and failure rate. The comparison examines whether the speed-quality-risk trade-off spectrum in the main text is retained on a second lens stack.

Supplementary Tables

Table S1. End-to-end latency statistics across the three platform types. Key quantiles of T_{pipe} are reported under the unified transaction-chain convention; T_{sa} is reported only where a comparable sampling reference is available.

Platform	Metric	Mean (ms)	p50 (ms)	p95 (ms)	p99 (ms)	Max (ms)	Comparable ref.
P1	T_{pipe}	0.00105	0.00105	0.00105	0.00105	0.00105	Yes
P1	T_{sa}	11.00105	11.00105	11.00105	11.00105	11.00105	Yes
P2	T_{pipe}	0.48090	0.41543	0.99840	1.54918	2.20627	Yes
P2	T_{sa}	11.48090	11.41543	11.99840	12.54918	13.20627	Yes
P3	T_{pipe}	119.46233	123.53731	243.86559	252.30454	272.14820	Yes
P3	T_{sa}	—	—	—	—	—	No

Table S2. Segment-wise latency statistics across the three platform types. The table lists the key quantiles of TS2−TS1, TS3−TS2, and TS4−TS3, together with dispersion statistics used in the tail-dominance analysis.

Platform	Segment	Mean (ms)	SD (ms)	p50 (ms)	p95 (ms)	p99 (ms)	p99 95% CI (ms)	Max (ms)
P1	TS2−TS1	0.0E+00	0.0E+00	0.0E+00	0.0E+00	0.0E+00	[0.0E+00, 0.0E+00]	0.0E+00
P1	TS3−TS2	1.0E-03	0.0E+00	1.0E-03	1.0E-03	1.0E-03	[1.0E-03, 1.0E-03]	1.0E-03
P1	TS4−TS3	5.0E-05	0.0E+00	5.0E-05	5.0E-05	5.0E-05	[5.0E-05, 5.0E-05]	5.0E-05
P2	TS2−TS1	0.302	0.252	0.231	0.787	1.336	[1.218, 1.426]	2.000
P2	TS3−TS2	0.070	0.048	0.060	0.161	0.226	[0.216, 0.244]	0.351
P2	TS4−TS3	0.109	0.098	0.079	0.301	0.479	[0.452, 0.508]	0.900
P3	TS2−TS1	4.816	4.523	2.750	14.408	20.415	[19.922, 21.115]	30.294
P3	TS3−TS2	0.947	0.695	0.816	1.598	3.975	[2.305, 5.744]	8.800

Platform	Segment	Mean (ms)	SD (ms)	p50 (ms)	p95 (ms)	p99 (ms)	p99 95% CI (ms)	Max (ms)
P3	TS4–TS3	112.867	74.632	118.432	235.278	242.307	[241.494, 243.114]	243.511

Table S3. Correspondence between P2 phenomenon-cluster reproduction and the main P3 phenomena.

Main phenomenon on P3	Controlled one-factor response on P2	Mechanistic meaning
Within the observable decomposition of T_{pipe}, the dominant tail of P3 is concentrated in TS4–TS3	As λ_{act} increases, $TTAF_{p99}$ and failure rate deteriorate simultaneously	One of the key sources of the dominant tail in the observable segment is uncertainty in the command-to-actuation path
Fixed rollback can improve the mean but is difficult to use for tail compression	Under constant compensation, improvement in mean Q is limited, while the heavy tail remains	Fixed rollback can in essence compensate only the average offset
No strategy is globally optimal	Strategy ranking is reordered when scanning σ_{align} and λ_{act}	The frontier should be understood as a trade-off spectrum under capability constraints

Table S4. Sensitivity of TS4–TS3 dominance within T_{sa} to the unobservable front-end offset Δ_{hidden} on P3. The scan uses $T_{sa} = \Delta_{hidden} + T_{pipe}$, with the p99 values of TS4–TS3 and T_{pipe} equal to 242.307 ms and 252.305 ms, respectively. The share column reports the p99 of TS4–TS3 divided by the p99 of T_{sa}; 95% CIs use B = 10,000 bootstrap resamples. Across $\Delta_{hidden} \in [1, 50]$ ms, the share remains above the 50% dominance threshold, with a tipping point near $\Delta_{hidden}^{*} \approx 232$ ms.

Δ_{hidden} (ms)	T_{sa} p99 (ms)	Share (%)	95% CI (%)	Above κ = 50%
0 (baseline)	252.30	96.04	[95.34, 96.89]	Yes
1 (lower bound)	253.30	95.66	[94.97, 96.50]	Yes
5	257.30	94.17	[93.50, 94.99]	Yes
10	262.30	92.38	[91.73, 93.16]	Yes
20	272.30	88.98	[88.39, 89.71]	Yes
30	282.30	85.83	[85.27, 86.52]	Yes
50 (upper bound)	302.30	80.15	[79.67, 80.77]	Yes
232 (tipping point)	484.30	50.00	—	Critical

Table S5. Scene-stratified variance measures for Q, TTAF, and failure rate on P3 (Lens A), reported for each (scene, strategy) cell (n = 120). Q_{mean} is reported with sample SD and a 95% Student's t-interval; $TTAF_{p50}$ and $TTAF_{p99}$ use 95% percentile bootstrap CIs (B = 10,000); failure rate uses a 95% Wilson score CI. Failure is defined as Q ≤ 0.95 or TTAF ≥ 5,000 ms. Scene abbreviations: HRD, high-texture random detail; LWB, low-texture/low-contrast broad peak; PRM, periodic repetitive-texture pseudo-multipeak; DTE, directional texture/single-edge; LHS, local highlight/specular-reflection disturbance; LHN, low-illumination high-noise.

Scene	Strategy	Q_{mean} (SD)	Q 95% CI	$TTAF_{p50}$ 95% CI (ms)	$TTAF_{p99}$ 95% CI (ms)	Failure rate	95% Wilson CI
HRD	S1	0.8848 (0.0168)	[0.8818, 0.8878]	[1040.7, 1095.3]	[1258.7, 1362.0]	100.00%	[96.91, 100.00]
HRD	S2	0.9652 (0.0267)	[0.9603, 0.9700]	[1704.1, 1752.6]	[1865.0, 1907.0]	19.17%	[13.10, 27.13]

Scene	Strategy	Q_{mean} (SD)	Q 95% CI	$TTAF_{p50}$ 95% CI (ms)	$TTAF_{p99}$ 95% CI (ms)	Failure rate	95% Wilson CI
HRD	S3	0.9744 (0.0198)	[0.9708, 0.9780]	[2351.8, 2457.6]	[2836.9, 3161.0]	9.17%	[5.19, 15.69]
HRD	S4	0.9941 (0.0024)	[0.9937, 0.9945]	[3438.0, 3504.0]	[3615.8, 3668.5]	0.00%	[0.00, 3.09]
HRD	S5	0.9948 (0.0043)	[0.9941, 0.9956]	[4347.9, 4390.8]	[4536.2, 4572.0]	0.00%	[0.00, 3.09]
LWB	S1	0.8580 (0.0270)	[0.8531, 0.8628]	[1115.8, 1187.9]	[1374.4, 1509.1]	100.00%	[96.91, 100.00]
LWB	S2	0.9489 (0.0352)	[0.9425, 0.9553]	[1794.7, 1860.9]	[1993.6, 2061.5]	25.83%	[18.82, 34.32]
LWB	S3	0.9645 (0.0263)	[0.9597, 0.9692]	[2457.5, 2584.4]	[2999.9, 3374.4]	10.83%	[6.44, 17.64]
LWB	S4	0.9897 (0.0042)	[0.9889, 0.9904]	[3544.4, 3629.1]	[3757.7, 3837.3]	0.00%	[0.00, 3.09]
LWB	S5	0.9870 (0.0067)	[0.9858, 0.9883]	[4652.5, 4743.0]	[4941.4, 5051.3]	4.17%	[1.79, 9.39]
PRM	S1	0.8669 (0.0249)	[0.8624, 0.8714]	[1087.6, 1151.8]	[1331.0, 1453.9]	100.00%	[96.91, 100.00]
PRM	S2	0.9574 (0.0319)	[0.9517, 0.9632]	[1780.9, 1842.9]	[1972.2, 2035.7]	12.50%	[7.73, 19.63]
PRM	S3	0.9663 (0.0245)	[0.9618, 0.9707]	[2436.4, 2559.0]	[2967.3, 3331.7]	10.00%	[5.80, 16.74]
PRM	S4	0.9878 (0.0048)	[0.9869, 0.9886]	[3544.4, 3627.6]	[3757.7, 3837.3]	0.00%	[0.00, 3.09]
PRM	S5	0.9698 (0.0119)	[0.9676, 0.9719]	[4696.0, 4798.0]	[5002.4, 5119.8]	10.83%	[6.44, 17.64]
DTE	S1	0.8743 (0.0209)	[0.8705, 0.8781]	[1068.8, 1129.2]	[1302.1, 1417.2]	100.00%	[96.91, 100.00]
DTE	S2	0.9575 (0.0309)	[0.9519, 0.9631]	[1765.5, 1824.8]	[1950.8, 2010.0]	21.67%	[15.22, 29.88]
DTE	S3	0.9675 (0.0260)	[0.9627, 0.9722]	[2457.5, 2584.4]	[2999.9, 3374.4]	12.50%	[7.73, 19.63]
DTE	S4	0.9912 (0.0038)	[0.9905, 0.9919]	[3526.7, 3607.0]	[3734.1, 3809.2]	0.00%	[0.00, 3.09]
DTE	S5	0.9813 (0.0118)	[0.9792, 0.9834]	[4609.0, 4692.7]	[4883.5, 4982.8]	4.17%	[1.79, 9.39]
LHS	S1	0.8551 (0.0273)	[0.8502, 0.8600]	[1078.3, 1140.5]	[1316.6, 1435.5]	100.00%	[96.91, 100.00]
LHS	S2	0.9513 (0.0337)	[0.9452, 0.9574]	[1765.5, 1824.8]	[1950.8, 2010.0]	17.50%	[11.73, 25.33]

Scene	Strategy	Q_{mean} (SD)	Q 95% CI	$TTAF_{p50}$ 95% CI (ms)	$TTAF_{p99}$ 95% CI (ms)	Failure rate	95% Wilson CI
LHS	S3	0.9649 (0.0232)	[0.9607, 0.9691]	[2425.8, 2546.3]	[2951.0, 3310.4]	7.50%	[4.00, 13.69]
LHS	S4	0.9864 (0.0048)	[0.9856, 0.9873]	[3526.7, 3607.0]	[3734.1, 3809.2]	0.00%	[0.00, 3.09]
LHS	S5	0.9732 (0.0091)	[0.9715, 0.9748]	[4565.5, 4642.4]	[4825.6, 4914.4]	0.00%	[0.00, 3.09]
LHN	S1	0.8502 (0.0298)	[0.8448, 0.8555]	[1134.6, 1208.3]	[1403.4, 1545.9]	100.00%	[96.91, 100.00]
LHN	S2	0.9412 (0.0388)	[0.9342, 0.9482]	[1827.0, 1897.0]	[2036.5, 2113.0]	32.50%	[24.81, 41.28]
LHN	S3	0.9567 (0.0316)	[0.9510, 0.9624]	[2499.9, 2635.1]	[3065.1, 3459.7]	17.50%	[11.73, 25.33]
LHN	S4	0.9829 (0.0106)	[0.9809, 0.9848]	[3615.3, 3709.9]	[3852.4, 3949.9]	2.50%	[0.85, 7.11]
LHN	S5	0.9670 (0.0157)	[0.9641, 0.9698]	[4823.6, 4919.8]	[5172.0, 5325.2]	19.17%	[13.10, 27.13]

Table S6. Compact physical calibration summary for the controlled perturbation levels.

Perturbation axis	Control levels	Compact calibration
σ_{align}	0, 1, 2, 3, 4, 5, 6 steps	$\sigma_{t,RMS}$ = 12.5 σ_{align} ms; endpoint: 75.0 ms at σ_{align} = 6.
λ_{act}	0.0, 0.2, 0.4, 0.6, 0.8, 1.0	TS4–TS3 p99 = 0.479 + 241.828 λ_{act} ms; endpoint: 242.307 ms at λ_{act} = 1.

Table S7. Goodness-of-fit between P2 one-factor perturbation scans and the P3 degradation pattern. Each condition is represented by a standardized degradation vector using five strategies and three metrics: failure rate, quality loss 1−Q_{mean}, and log($TTAF_{p99}$). Smaller nRMSE and nMAE indicate closer magnitude agreement; larger Cosine, Pearson r, Kendall τ, and Spearman ρ indicate stronger agreement in direction, component pattern, or strategy-risk ranking. Rows marked as best bridge have the minimum nRMSE within the corresponding one-factor scan. At zero injection, direction and correlation metrics are not applicable because the P2 degradation vector is zero.

Axis	Intensity	Point type	nRMSE↓	nMAE↓	Cosine↑	Pearson r↑	Kendall τ↑	Spearman ρ↑
σ_{align}	0 step	scan level	2.0273	1.5891	—	—	—	—
σ_{align}	1 step	scan level	1.7663	1.3844	0.9931	0.9824	1.000	1.000
σ_{align}	2 step	scan level	1.4610	1.1423	0.9942	0.9850	1.000	1.000
σ_{align}	3 step	scan level	0.9245	0.7185	0.9965	0.9909	1.000	1.000
σ_{align}	4 step	scan level	0.5419	0.4162	0.9979	0.9945	1.000	1.000
σ_{align}	5 step	scan level	0.2828	0.2108	0.9991	0.9976	0.800	0.900
σ_{align}	6 step	best bridge	0.0256	0.0118	0.9999	0.9998	1.000	1.000
λ_{act}	0.0	scan level	2.0474	1.6062	—	—	—	—
λ_{act}	0.2	scan level	1.5738	1.2349	0.9893	0.9732	1.000	1.000

Axis	Intensity	Point type	nRMSE↓	nMAE↓	Cosine↑	Pearson r↑	Kendall τ↑	Spearman ϱ↑
λ_{act}	0.4	scan level	1.0628	0.8262	0.9887	0.9713	1.000	1.000
λ_{act}	0.6	scan level	0.6732	0.5128	0.9929	0.9817	1.000	1.000
λ_{act}	0.8	scan level	0.3711	0.2632	0.9955	0.9883	1.000	1.000
λ_{act}	1.0	best bridge	0.0244	0.0113	0.9999	0.9998	1.000	1.000
